# Erratum: Dendritic cell derived exosomes loaded with immunoregulatory cargo reprogram local immune responses and inhibit degenerative bone disease *in vivo*

**DOI:** 10.1002/jev2.12115

**Published:** 2021-07-22

Elashiry, M., Elashiry, M.M., Elsayed, R., Rajendran, M., Auersvald, C., Zeitoun, R., Rashid, M.H., Ara, R., Meghil, M.M., Liu, Y., Arbab, A.S., Arce, R.M., Hamrick, M., Elsalanty, M., Brendan, M., Pacholczyk, R. and Cutler, C.W. (2020), Dendritic cell derived exosomes loaded with immunoregulatory cargo reprogram local immune responses and inhibit degenerative bone disease *in vivo*. Journal of Extracellular Vesicles, 9: 1795362. https://doi.org/10.1080/20013078.2020.1795362

The authors inadvertently left the following grant information out of the Acknowledgments section of their article:

The MicroCT data shown in Figure 10 was made possible through the support of an NIH S10 grant (S10 OD025177).

The authors regret the error.

